# Entrepreneurial Leadership and Turnover Intention of Employees: The Role of Affective Commitment and Person-job Fit

**DOI:** 10.3390/ijerph16132380

**Published:** 2019-07-04

**Authors:** Juan Yang, Bo Pu, Zhenzhong Guan

**Affiliations:** 1 School of Economics and Management, Southwest Jiaotong University, Chengdu 610031, China; 2 College of Tourism, Sichuan Agricultural University, Chengdu 611830, China

**Keywords:** Entrepreneurial leadership, Person-job fit, Affective commitment, Turnover intention

## Abstract

The purpose of this study is to explore the causal system between entrepreneurial leadership and turnover intention of employees by examining the mediating effect of affective commitment and the moderating effect of person-job fit in start-ups. A quantitative approach was used to test the hypotheses and data were collected through the internet questionnaire tool. The authors selected employees from ventures newly established within the past five years and finally collected 427 questionnaires. The authors then used the hierarchical regression analysis method of Baron and Kenny for test mediating effect and the Hayes bootstrapping method for the test moderating effect by using Hayes’ SPSS PROCESS macro. The results demonstrated that affective commitment functions as a mediator and person-job fit functions as a moderator. This paper provides implications for start-up leaders that entrepreneurial leadership is an effective style of leadership and plays a crucial role which accompanies the development of venture.

## 1. Introduction

In the Amway Global Entrepreneurship Report 2018, which describes a study examining the attitudes towards entrepreneurship among 44 countries worldwide, China was found to have a higher potential for entrepreneurship than average, with 86% of citizens expressing positive attitudes towards entrepreneurship and China now ranking third in terms of Amway Entrepreneurial Spirit Index (AESI) (incorporating three equally weighted dimensions: desire, feasibility and stability) behind Vietnam and India [1]. Nevertheless, the high turnover intention of employees has become a widespread problem accompanying the development of start-ups according to the survey report of 2018 turnover and a salary survey report by 51job.com [2]. In previous research, some studies have demonstrated that the behavior of leaders has a distinct impact on the turnover intention of employees, including servant leadership [3], transactional leadership, affective leadership [4], ethical leadership [5], transformational leadership [6,7] and authentic leadership [8]. However, little research has been performed to examine entrepreneurial leadership.

Entrepreneurial leadership (EL), as an intersection of entrepreneurship and leadership [9], highlights the entrepreneurial behavior and capability a leader shows in reaction to dynamic changes. For example, to recognize and discover entrepreneurial opportunities [10,11] and to explore and exploit strategic value creation [12] are both essential qualities of entrepreneurial leadership. Entrepreneurial leadership refers to a leading role in a start-up business specifically, rather than merely an entrepreneurial style of leadership which can be applied in both mature and start-up ventures [13,14]. Under the current entrepreneurial environment, the majority of ventures are facing a more uncertain business environment and a more competitive market. Therefore, obtaining an in depth understanding of the impact of entrepreneurial leadership on the turnover intention of employees will extend the scope of future study in this field and can guide leaders towards more effective mechanisms to start and grow businesses.

**Figure 1:**
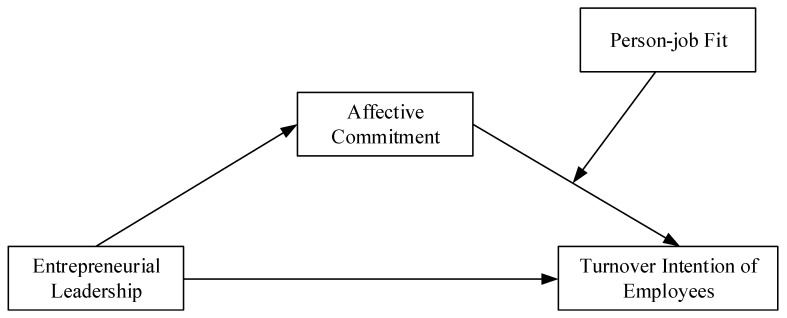
Conceptual model.

This study concentrates on two crucial aspects in newly established ventures. First, it explores the indirect effect of entrepreneurial leadership on the turnover intention of employees through affective commitment, revealing the relationship from the perspective of affective commitment. Second, it studies the moderating effect of person-job fit on the relationship between affective commitment and turnover intention of employees, revealing the boundary condition of the entrepreneurial leadership function. The conceptual model of this study is shown in Figure 1.

## 2. Theory and Hypotheses

### 2.1. Entrepreneurial Leadership

Entrepreneurial leadership is considered to be the intersection of the fields of entrepreneurship and leadership [9]. Based on the research on entrepreneurship by McGrath and MacMillan [15], Gupta et al. [12] proposed that entrepreneurial leadership is the behavior of leaders that inspires employees to discover and create strategic value by initiating visions, mobilizing employees and establishing commitments. This group of scholars also proposed a general structure model for entrepreneurial leadership, incorporating two dimensions, scenario enactment and cast enactment, and five roles, framing the challenge, absorbing uncertainty, path-clearing, building commitment and specifying limits. They suggested that the two critical goals that entrepreneurial leadership should meet are 1) creating opportunities and improving status quo with limited resources; and 2) persuading potential followers and stakeholders to accomplish objectives by integrating resources, including recruiting extra personnel. The two enactments are interdependent. Without an appropriate cast, no scenario enactment can be devised. Simultaneously, only with a convincing scenario can a cast enactment be realized. After comparing the characteristics of entrepreneurial leadership with those of leadership, Tarabishy et al. [11] proposed that entrepreneurial leadership should encompass five dimensions: establishing visions, resolving problems, decision-making, risk-taking and strategic policies. After surveying 61 medium-small sized ventures in manufacturing and service industries, Hejazi, Malei, and Naeiji [16] proposed four dimensions to evaluate entrepreneurial leadership: strategic, communicative, personal and motivational dimensions.

Compared with charismatic leaders and transformational leaders, entrepreneurial leaders encounter a highly uncertain business environment while leading employees. However, due to the emphasis on personality, charismatic and transformational leaders often marginalize the characteristics of employees [17], whilst entrepreneurial leaders are considered to be anti-heroism proponents. Vecchio [18] introduced a model which integrates process issues and level issues, and analyzed the process dynamics of entrepreneurial leaders in different phases of firm start-up, from both macro- (contextual) and micro- (psychological) perspectives. In different phases of firm start-up, both macro- and micro- factors mutually influenced entrepreneurial leadership and eventually affected behaviors of employees. During the prelaunch and launch phases of firm start-up, entrepreneurs should equip themselves with the entrepreneurial Big Five: risk-taking propensity, need for achievement, need for autonomy, self-efficacy and locus of control [19,20]. With the Big Five, accompanied by social capital, entrepreneurial opportunities and support, entrepreneurs can attain resources and develop opportunities to achieve entrepreneurial success [21]. These features were important in the early phase of firm start-ups, as previous scholars have described [22,23,24]. Nevertheless, most empirical studies on entrepreneurial leadership do not take the early phase of firm into account. Instead, studies have shown great interest in the relationship between entrepreneurial leadership and other aspects of business and management. For example, previous studies have been concerned with the relation between entrepreneurial leadership and innovative behavior of employees [25], venture performance [26,27], job satisfaction of employees [28] and international human resource management and global competition advantage [29]. Some other studies concerned entrepreneurial leadership in mid-sized family firms [30,31] and the role of gender in leadership [32,33]. However, there is little research on the relationship between entrepreneurial leadership and employee turnover intentions in the initial stage of entrepreneurship.

### 2.2. Entrepreneurial Leadership and Turnover Intention of Employees

The research on turnover intention was initially started from the perspective of March and Simon [34], which refers to the possibility of an employee voluntarily leaving the company within a certain period of time [35] Several models of voluntary turnover were subsequently introduced, including Mobley’s model of the turnover process in multiple stages [36], Steers–Mowday’s model of turnover [37], Price’s model of turnover [38] and Lee and Mitchell’s unfolding model of employee turnover [39]. These models encompass two major elements with regard to work attitudes, 1) job satisfaction and organizational commitments; and 2) the mobility of employee turnover, which is illustrated in the awareness of alternative jobs and the process of job hunting [40].

Voluntary turnover of employees often results in great loss of human and social capital, leading to a negative repercussion for organizations [41]. When employees leave firms, they take not only their individual intelligence, experience and skills, but also relative resources and social capital. A lot of empirical research has proved that turnover intention has a positive relationship with actual turnover, and it can be used as a valid alternative for actual turnover behavior [42].

A review of the relevant literature indicates that the majority of studies are concerned with the relationship between various styles of leadership (e.g., transformational leadership, ethical leadership, authentic leadership) and turnover intention [8,43,44]. For example, empirical findings by Gyensare et al. [43] showed a positive association between transformational leadership and turnover intention. Azanza et al. [8] used structural equation modelling to test the mechanism between authentic leadership and the employees’ turnover intentions.

This study positions entrepreneurial leaders as pivotal figures in a venture. Through initiating visions, mobilizing employees and obtaining commitment from them, entrepreneurial leaders are capable of uniting employees, resulting in a low turnover ratio. Based on this logic, this study proposes the following hypothesis.

**Hypothesis 1 (H1)**: Entrepreneurial Leadership has a negative impact on the turnover intention of employees.

### 2.3. Mediating Role of Affective Commitment

The affective component of organizational commitment reflects one’s liking of the job and emotional attachment to the enterprise [39]. Prior research suggested that individual characteristics, organizational characteristics and job-related characteristics were the antecedents of affective commitment [45]. Leadership is one of the organizational factors that is considered a key determinant of organizational commitment. Jackson, Meyer, and Wang [46] suggested that transformational/charismatic leadership was positively associated with affective commitment. Entrepreneurial leaders influence subordinates’ affective commitment by framing an achievable challenge, and inspiring a commitment to work hard.

Also, affective commitment is the most accepted dimension of the three aspects of organizational commitment which contained affective, continuance, and normative commitment, because it is considered the most consistent and powerful antecedent variable of turnover intentions [47,48]. Joarder, Sharif, and Ahmmed [49] examined the connection between affective commitment and turnover intention, and showed that affective commitment has negatively and significantly impact on turnover intention. Consistent with previous studies, A’Yuninnisa and Saptoto [50] examined the link between pay satisfaction and turnover intention through affective commitment. It was found that pay satisfaction had a direct and indirect effect via affective organizational commitment on turnover intention. Slugoski [51] explored whether job embeddedness, job satisfaction, and organizational commitment influence employee retention. The results showed that organizational commitment had the largest impact on intent to stay, followed by job satisfaction, and job embeddedness. From the above, this study proposes the following hypothesis.

**Hypothesis 2 (H2)**: Entrepreneurial Leadership and turnover intention is mediated by affective commitment rather than direct relationships.

### 2.4. Moderating Role of Person-job Fit

Edwards [52] defined person-job fit as the match between personal skills and vocational requirements, or as the match between personal requirements and vocational characteristics. Person-job fit indicates the correspondence between job reinforcement system and need system of the individual [53]. Hence, person-job fit is an important concept both for employees and recruiters. A considerable amount of research has described positive correlations between person-job fit, commitment to the organization and the success of the vocation, respectively [54,55]. Simultaneously, negative correlations have been observed between person-job fit and both turnover intention and turnover behavior [56,57].

Compared with mature ventures, Start-up businesses usually employ relatively younger workers [58] who are likely to switch jobs early on in their careers as they learn about their own skills [59]. Therefore, the leaders of start-ups need to more concern about the employees’ affective need. In our study, the data is collected from private enterprises which started in the last five years. We speculate that a higher person-job fit suggests higher personal initiative and these employees will make better use of their abilities, thus benefiting the fulfilment of individual objectives [52], and with less concern about affective commitment. On the contrary, employees with lower person-job fit may have problems coordinating with colleagues and cannot take advantage of their own strengths, thus these employees may pay more attention to affective commitment. Based on the argument described above, this study proposes the following hypothesis.

**Hypothesis 3 (H3)**: Person-job fit moderates the negative relationship between affective commitment and turnover intention; the higher the fit, the weaker the negative correlation between affective commitment and turnover intention.

Based on the arguments above, this study postulates that 1) affective commitment is a mediator between entrepreneurial leadership and turnover intention of employees; and 2) person-job fit weakened the negative impact of affective commitment on employees’ turnover intention, but has no effect on the negative correlation between entrepreneurial leadership and the affective commitment of employees. According to these hypotheses, it can be postulated that a higher person-job fit suggests a weaker negative influence of affective commitment on the turnover intention of employees. In other words, a higher person-job fit indicates a weak indirect effect of entrepreneurial leadership on turnover intention of employees through affective commitment.

**Hypothesis 4 (H4):** The higher the employee’s degree of person-job fit, the weaker the mediating effect of affective commitment between entrepreneurial leadership and turnover intention of employees.

## 3. Method

### 3.1. Sample and Procedures

Data for this study were collected through an online survey agency, which is Wenjuanxing. We made clear to the agency that the respondents should be from ventures newly established within the past five years and restricted the IP access, controlled answer time in order to avoid repeating questionnaires and improving the quality of questionnaires. Each business had no more than 10 respondents, in total, 500 questionnaires were collected from 62 ventures, with 427 deemed valid after the elimination of those without basic employee information. The sample originated from major cities and provinces across China, including Shanghai, Beijing, Guangdong, Jiangsu, Shandong, Sichuan and Hubei. The majority of participants were female (63.7%). Most participants were aged between 21 and 35 years (87.4%). In terms of education, 69.1% of participants had a bachelor’s degree, 23% had associate college degrees and 7.9% had master’s degrees. Most companies had fewer than 100 employees (75.2%).

### 3.2. Measures

All measurement scales applied in this study are tools widely used in relevant research, with necessary adjustments for this particular study (see Appendix A).

Entrepreneurial leadership (EL). Huang [60] derived 26 items for measuring EL based on the scale by Gupta et al. [12] who designed 19 items from the Global Leadership and Organizational Behavior Effectiveness (GLOBE) study. This measure includes five dimensions: framing challenge, absorbing uncertainty, path-clearing, building commitment and specifying limits. Each item was measured on a 7-point Likert type scale ranging from 1 (strongly disagree) to 7 (strongly agree). An exploratory factor analysis was first conducted, the dimension of framing challenge was deleted and 17 items were reserved with four dimensions. Sample items included: “company leaders tend to set high standards of performance”, “company leaders have strong foresight and grasp of the company’s development prospects” and “leaders can inspire employees to achieve the company’s goals”. The Cronbach’s alpha for this scale was 0.89.

Turnover intention of employees. A three-item single-dimension scale by Liang [61] was applied. These items were measured on a 7-point Likert type scale ranging from 1 (strongly disagree) to 7 (strongly agree). Items included: “I often want to leave this company”, “I will probably find a new job next year” and “Recently, I often want to change my job”. The Cronbach’s alpha for this scale was 0.88.

Affective Commitment. Yao, Huang, and Fan [62] derived four items for measuring affective commitment, based on the scale developed by Allen and Meyer [63] and Ko, Price, and Mueller [64]. Items were measured on a 7-point Likert type scale ranging from 1 (strongly disagree) to 7 (strongly agree). Items included “I’m glad to work in this company”, “I feel like I’m part of this company”, “I feel a sense of belonging in this company” and “I have a deep affection for this enterprise”. The Cronbach’s alpha for this four-item scale was 0.88.

Person-job fit. A four-item single-dimension scale by Weng and McElory [65], which was adjusted based on Singh and Greenhaus [66], was applied. Items were measured on a 7-point Likert type scale ranging from 1 (strongly disagree) to 7 (strongly agree). Items included: “I feel matching with my current job very much”, “The requirements of my present job are in line with my experience, skills and knowledge”, “The working environment provided by the company is in line with my requirements” and “My personality and temperament match my work”. The Cronbach’s alpha for this scale was 0.77.

Control variables. Previous research indicates that employee demographics (e.g., age, salary, number of people in the venture and corporate tenure) have a noticeable influence on turnover intention [38,67]. Therefore, this study treated the following variables as control variables: employee age, employee salary, number of people in the venture and corporate tenure.

### 3.3. Data Analysis

Previous studies generally follow hierarchical regression analysis method of Baron and Kenny [68] for the test of mediation. Another way of examining mediation is through the bootstrapping method, which was initially introduced by Preacher and Hayes [69], then recommended by Zhao, Gao, and Wang [70], and has been widely applied in the field of organizational behavior (OB). This study used AMOS 24.0 and PROCESS macro for SPSS 24.0 to test the hypotheses.

## 4. Results

### 4.1. Confirmatory Factor Analysis (CFA)

In order to examine the measurement parameters of each scale and the discriminate validity among the four crucial variables, “entrepreneurial leadership”, “affective commitment”, “person-job fit” and “turnover intention”, this study used AMOS 24.0 to conduct CFA on the four crucial variables and to compare outcomes among four-, three-, two- and single factor(s) models. The results indicated that both the CFI and TLI were greater than 0.90 in the four-factor model, whilst the RMSEA was smaller than 0.08. The analytical statistics corroborate the observed data (χ2 = 689.87, *p* < 0.01; RMSEA = 0.05, CFI = 0.93, TLI = 0.93), shown in Table 1, suggesting the discriminate validity of the measurements.

### 4.2. Descriptive Statistics

Table 2 summarizes the mean, standard deviation and correlations of the variables. There was a strong positive correlation between entrepreneurial leadership and affective commitment (r = 0.64, *p* < 0.01) and a strong negative correlation between entrepreneurial leadership and turnover intention (r = –0.50, *p* < 0.01). Among the control variables, employee age, monthly salary, number of employees and corporate tenure all had strong negative correlations with turnover intention.

### 4.3. Hypothesis Testing

According to Baron and Kenny’s [68] criterion, there are three conditions for the existence of mediating role: 1) there is a significant correlation between independent variable (entrepreneurial leadership) and dependent variable (turnover intention); 2) there is a significant correlation between independent variable (entrepreneurial leadership) and mediating variables (affective commitment); 3) finally, the regression coefficients of independent variables (entrepreneurial leadership) and mediating variable (affective commitment) are simultaneously regressed to the dependent variable (turnover intention), the coefficient of mediator should be significant and the coefficient of independent variable become non-significant (complete mediation) or reduced (partial mediation).

According to these three steps, the outcomes of the hierarchical regression analysis are listed in Table 3. It can be seen that there was a strong negative relationship between entrepreneurial leadership and turnover intention (M4, β = –0.48, *p* < 0.05). Therefore, the first condition is satisfied. Hence, hypothesis 1 (H1) is accepted with the support of the analytical statistics.

After the addition of the mediating variable (affective commitment), entrepreneurial leadership still had a negative effect on turnover intention, although there was a smaller regression coefficient (M6, β = –0.14, *p* < 0.01), from −0.48 to −0.14. Therefore, we can conclude that affective commitment has a partial mediating effect on the relationship between entrepreneurial leadership and turnover intention of employees. Hence, hypothesis 2 (H2) is partially accepted.

**Figure 2:**
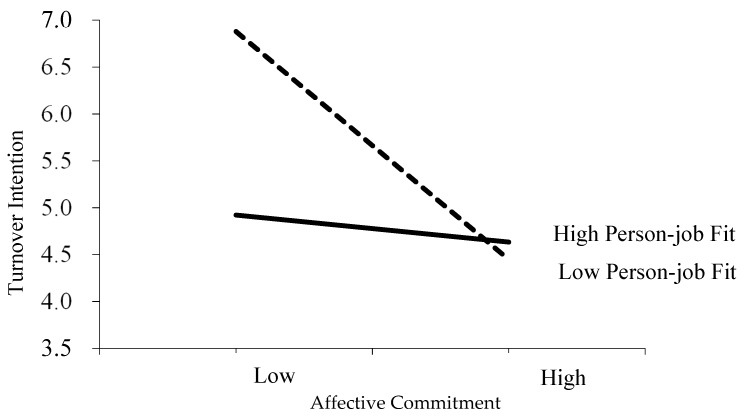
Influence of entrepreneurial leadership on affective commitment under different person-job fit scenarios.

To examine the moderation and the mediated moderation predicted by Hypotheses 3 and 4, respectively, we adopted the moderated causal step approach of analysis [71]. Five steps were carried out: first, set turnover intention as the dependent variable and then added the control variables, the independent variable (entrepreneurial leadership), the mediating variable (affective commitment), the moderating variable (person-job fit) in the second, third and fourth step. Finally, the multipliers of affective commitment and person-job fit interaction were entered. To eliminate the linearity, both the mediating variable and the moderating variable were standardized when constructing their multipliers [72]. The results of the regression analysis are listed in Table 3, which shows the strong positive correlation between turnover intention and the interaction of affective commitment and person-job fit (M8, β = 0.52, p < 0.05). The results indicate that the higher the fit, the weaker the negative correlation between affective commitment and turnover intention. Hence, hypothesis 3 (H3) is accepted. Figure 2 demonstrates the interactive effects of affective commitment and turnover intention. Based on the procedure recommended by Cohen, J., Cohen, P., West, and Aiken [73], we visualize the influence of affective commitment on turnover intention with two different person-job fit scenarios: one standard deviation higher and one standard deviation lower than the average.

Hypothesis 4 proposes that the indirect effect of entrepreneurial leadership on the turnover intention of employees through affective commitment is contingent on person-job fit. To examine this hypothesis, this study applied model 14 of Hayes’ SPSS PROCESS macro [74] to analyze the indirect effect of affective commitment under different degrees of person-job fit; the results are listed in Table 4 and Table 5. The findings show that the indirect effect of entrepreneurial leadership on the turnover intention of employees through affective commitment becomes weaker as the level of person-job fit increases. The mediating role of affective commitment between entrepreneurial leadership and turnover intention of employees was significant for low (b = –0.61, SE = 0.08, 95% CI = [−0.77, −0.44]), middle (b = –0.53, SE = 0.08, 95% CI = [−0.70, −0.37]) and high levels of person-job fit (b = –0.45, SE = 0.10, 95% CI = [−0.64, −0.26]). Therefore, the proposed moderated mediation hypothesis (H4) is supported. That is, the moderated mediation analysis suggests that the higher the person-job fit, the weaker the mediating role of affective commitment between entrepreneurial leadership and turnover intention of employees.

## 5. Discussion

In general, when studying factors affecting turnover intention of employees, both domestic and global researchers have concentrated less on the perspective of entrepreneurial leadership and more on that of behavioral characteristics of leaders. Results from prior research shows that transformational leadership is strongly negatively correlated with turnover intention [8,43,44]. For example, empirical findings by Gyensare et al. [43] showed a positive association between transformational leadership and turnover intention. Demirtas and Akdogan [44] found that the behavior of ethical leadership could influence awareness of ethical climate, which in sequence positively influenced employees’ turnover intentions. Azanza et al. [8] used structural equation modelling to test the mechanism between authentic leadership and the employees’ turnover intentions. Compared with other styles of leadership, we examined the impact of entrepreneurial leadership on turnover intentions of employees. Although there is some literature exploring how entrepreneurial leadership influences outcomes in mature enterprises [75], little field research has been done on entrepreneurial leadership in start-ups. One of our objectives was to address this gap by examining how entrepreneurial leadership impact employees’ attitude. The results support the contention that active leadership negatively affects turnover intention which is consistent with those of Wu [76]. The results also suggest that affective commitment mediates the relationship between entrepreneurial leadership and turnover intention.

There were sufficient studies to examine the person-environment fit which contained four critical domains of person-environment fit: person-job, person-organization, person-group, and person-supervisor fit [77]. For example, Alniaçik, Alniaçik, Erat, and Akçin [78] revealed that the level of person organization fit significantly moderates the effects of job satisfaction on turnover intentions. In this paper, we found that person-job fit moderates the relationship between affective commitment and turnover intention. Employees with higher person-job fit can better fulfil their vocational requirements [79], complete tasks more efficiently and produce better work performance, so they are less affected by affective commitment. The findings reported that leaders should more concentrate on commitment in order to reduce turnover, otherwise, when the commitment is low leaders should increase the degree of person-job fit.

### 5.1. Theoretical Implications

As far as we know, this study is the first try of a second-stage moderated mediation model for the relationship between entrepreneurial leadership and turnover intention. Although there has been a lot of research on the relationship between turnover intentions and various styles of leadership, but few researches focus on this relationship. So, this paper fills this gap. First, entrepreneurial leadership has a positive effect on affective commitment. Second, affective commitment has a negative effect on turnover intention, and lastly, person-job fit moderates the relationship between affective commitment and turnover intention. These findings suggest that this is a typical second-stage moderated mediation model. Exploring the moderated mediation relationships between entrepreneurial leadership and turnover intentions is not merely important for understanding the mechanism between them, but it also represents a milestone toward reducing turnover in start-ups.

This study has three contributions. First, we develop a model to explain the effectiveness of entrepreneurial leadership. The model predicts that entrepreneurial leadership decreases turnover intention. Second, the model provides understanding of the mediating role that affective commitment plays between entrepreneurial leadership and turnover intention. The findings support our hypothesis that entrepreneurial leaders influence subordinates’ turnover intention through affective commitment. Employees leave their job because of a lack of emotional restraint, which is caused by their leader. Finally, the study reinforces the idea that individual differences affect the behavioral outcomes. As Verquer, Beehr, and Wagner [56] point out, high level of person-job fit will decrease turnover intention, thus, highly person-job fit individuals are less likely to be concerned about emotional factors and reducing the negative effect of affective commitment on turnover intention.

### 5.2. Managerial Implications

Nowadays, small- and medium-sized enterprises account for a large share of total enterprises and make a huge contribution to real GDP growth and employment [80]. Entrepreneurial leadership plays a significant role in start-ups, especially in the current climate where enthusiasm for entrepreneurship continues to increase. The results of this research have important practical significance for start-ups. First of all, this study helps leaders in start-ups more thoroughly understand the characteristics and behaviors of entrepreneurial leaders. Further, it illustrates the process of how entrepreneurial leadership impact followers’ turnover intention. Through an indirect path (entrepreneurial leadership → affective commitment → turnover intention), we can judge that entrepreneurial leaders should pay more attention on followers’ affective commitment, which is an important variable in differentiating between the stayers and the leavers.

The results also show that employees with high job matching are less likely to be negatively influenced by affective commitment. Therefore, enterprises should pay more attention to putting the right person into the right position. Enterprises should pay special attention to employees who cannot meet the requirements of the post for the time being, and provide them with more training related to corporate culture and professional skills so that these employees can better adapt to the requirements of the current post and thus reduce the possibility to turnover.

### 5.3. Limits and Prospects

This study explored the process by which entrepreneurial leadership impacts on turnover intention, and the significant mediators and moderators of this process. The analyses applied not only traditional methods such as regression analysis, but also advanced methods such as bootstrapping using the SPSS PROCESS macro [74]. This detailed analysis profoundly benefits the depth and validity of this study by precisely depicting the relationships among the variables (e.g., direct effect, indirect effect and moderated mediation).

Nevertheless, due to various subjective and objective constraints, this study has the following inevitable limitations. First, we utilized the scale of entrepreneurial leadership adapted by Huang [60], which derived from Gupta et al. [12]. Even though the reliability and validity statistics for all variables in this study were deemed acceptable, a specialized scale addressing the actual conditions in China is unarguably necessary for future research. Second, we adopted a single cross-sectional study to get data, but the impact of entrepreneurial leadership on turnover intentions among start-ups enterprises should be examined longitudinally. Future research can collect data at different points in time to provide additional support for model causality. Third, we analyzed the influencing mechanism of entrepreneurial leadership from the perspective of employees, and our research can develop a self-assessment tool for leaders to evaluate their own EL. Fourth, although each business has no more than 10 respondents, the common method variance still cannot be avoided. Future research can collect data from more companies. Lastly, this study only investigated the contingency impact of person-job fit on the relationship between affective commitment and turnover intention. Factors other than person-job fit may also have similar influences, such as person–organization fit, person–group fit, and person–supervisor fit. These scenarios also have the potential to be examined in the future.

## 6. Conclusions

The purpose of this study is to explore the causal system between entrepreneurial leadership and turnover intention of employees by examining the mediating effect of affective commitment and the moderating effect of person-job fit in start-ups. The results demonstrated that affective commitment functions as a mediator and person-job fit functions as a moderator. As it is a rather new field, research on entrepreneurial leadership is at the exploratory stage. This study selected 427 employees from newly established ventures to form a sample to illustrate both the mediating effect of effective commitment and the moderating effect of person-job fit between entrepreneurial leadership and turnover intention of employees. The results indicate that 1) entrepreneurial leadership has a negative impact on turnover intention, with effective commitment functioning as a mediator; and 2) effective commitment has a negative impact on turnover intention, with person-job fit functioning as a moderator, weakening this negative relationship.

## Figures and Tables

**Table 1:** Confirmatory factor analysis results.

Model	χ2	df	TLI	CFI	RMSEA	AIC
Four-factor	689.87	344	0.93	0.93	0.05	813.87
Three-factor	745.90	347	0.91	0.92	0.05	863.90
Two-factor	948.43	349	0.87	0.88	0.06	1063.43
Single-factor	1474.62	350	0.76	0.78	0.09	1586.62

Note: *n* = 427; the single factor model integrates all variables into a single-factor; the two-factor model considers entrepreneurial leadership as a single factor and integrates turnover intention, affective commitment and person-job fit into one factor; the three-factor model considers entrepreneurial leadership and turnover intention as two single factors, while integrating affective commitment and person-job fit into one factor; the four-factor model considers all four variables as independent factors.

**Table 2:** Descriptive statistics and correlations for the main variables.

Variable	1	2	3	4	5	6	7	8
1 Employee Age								
2 Monthly Salary	0.29 **							
3 Number of Employees	0.15 **	0.29 **						
4 Corporate Tenure	0.41 **	0.31 **	0.32 **					
5 Entrepreneurial Leadership	0.11 *	0.20 **	0.12 *	0.23 **				
6 Affective Commitment	0.14 **	0.25 **	0.19 **	0.35 **	0.64 **			
7 Turnover Intention	−0.18 **	−0.29 **	−0.15 **	−0.27 **	−0.50 **	−0.69 **		
8 Person-job Fit	0.21 **	0.25 **	0.16 **	0.34 **	0.56 **	0.68 **	−0.59 **	
Mean	2.26	2.91	2.72	2.67	5.21	5.10	2.98	5.22
Standard Deviation	1.07	1.05	0.94	1.04	0.74	1.24	1.51	1.03

Note: Employee Age: “1”—21 to 25 years old; “2”—26 to 30 years old; “3”—31 to 35 years old; “4”—36 to 40 years old; “5”—41 years old and above. Monthly Salary: “1”—¥3000 and below; “2”—¥3001 to ¥5000; “3”—¥5001 to ¥8000; “4”—¥8001 to ¥15,000; “5”—above ¥15,000. Number of Employees: “1”—below 10 persons; “2”—10 to 50 persons; “3”—50 to 100 persons; “4”—100 persons above. Corporate Tenure: “1”—below 1 year; “2”—1 to 2 years; “3”—2 to 3 years; “4”—3 to 5 years. *n* = 427; * *p* < 0.05 (two-tailed), ** *p* < 0.01 (two-tailed).

**Table 3:** Results of the hypothesis testing.

	Affective Commitment		Turnover Intention					
	M1	M2	M3	M4	M5	M6	M7	M8
Control Variables								
Age	0.15 **	0.07	–0.22 **	−0.15 **	−0.12 **	−0.11 **	−0.11 **	−0.10 **
Monthly Salary	0.06	0.05	−0.03	−0.02	0.01	0.01	0.01	0.01
Number of Employees	0.31 **	0.19 **	−0.17 **	−0.08	0.03	0.03	0.04	0.04
Corporate Tenure	−0.04 **	−0.03	−0.05	−0.05	−0.07	−0.07	−0.06	−0.06
Independent Variable								
Entrepreneurial Leadership		0.58 **		−0.48 **		−0.14 **	−0.11 *	−0.12 **
Mediating Variable								
Affective Commitment					−0.66 **	−0.57 **	−0.48 **	−0.79 **
Moderating Variable								
Person-job Fit							−0.19 **	−0.43 **
Interaction								
Affective Commitment × Person-job Fit								0.52 *
R^2^	0.15	0.46	0.12	0.33	0.50	0.51	0.53	0.53
F-value	18.76 **	71.51 **	14.73 **	42.06 **	82.46 **	72.23 **	66.10 **	58.82 **
△R^2^	0.15	0.31	0.12	0.21	0.37	0.18	0.02	0.01
△F	18.76 **	240.05 **	14.73 **	132.96 **	310.19 **	149.11 **	14.92 **	4.26 *

Note: *n* = 427; ** *p* < 0.01, * *p* < 0.05. Dependent variable of M1, M2 is affective commitment, and dependent variable of M3 to M8 is turnover intention.

**Table 4:** Mediation model: mediating role of affective commitment between entrepreneurial leadership and turnover intention.

	b	SE	Bootstrap 95% CI
Indirect Effect	−0.69	0.07	[−0.84, −0.55]
Direct Effect	−0.29	0.08	[0.00, −0.45]

Note: Model 4 in the PROCESS macro. Bootstrap resample = 5000. b is the unstandardized regression coefficient, SE is standard error, CI is confidence interval.

**Table 5:** Analysis of moderated mediation.

Mediator	Conditional indirect effects of person-job fit			
	Condition	b	SE	Bootstrap 95% CI
Affective commitment	Low	−0.61	0.08	[−0.78, −0.45]
	Middle	−0.53	0.08	[−0.70, −0.37]
	High	−0.45	0.10	[−0.64, −0.26]

Note. Model 14 in the PROCESS macro. Bootstrap resample = 5000. Conditions for person-job fit are the mean plus or minus one standard deviation from the mean. Affective commitment and person-job fit were mean-centered prior to analysis.

## Appendix A. Adapted Scale of Construct.

**Construct**	**Scale reference**	**Adapted scale**	**Notes**
Entrepreneurial leadership	Huang et al. (2014)	Leaders prefer to set high standards for business performance	Deleted
		Leaders pursue continuous business performance improvement	Deleted
		Leaders adjust goals according to employee ability	Deleted
		Leaders draft long-term strategic goals of the company with the premise of understanding the market	Deleted
		Leaders are prone to set challenging goals	Deleted
		Leaders have concrete planning for future development to reduce uncertainty in the process	
		Leaders have strong predictability and control over the development prospect of the company	
		Leaders voluntarily and actively take the business risks to reduce uncertainty for employees in work	
		Leaders reduce uncertainty by various means to establish employee’s confidence in accomplishing the tasks	
		Leaders try to reduce the negative responses employees have during business transformation, such as the fear of uncertainty and concerns of failure, to the greatest extent	
		Leaders often communicate with employees regarding future development to reduce employee aversion to business transformation	
		Leaders have strong persuasiveness and can easily convince others and gain support	
		Leaders anticipate and eliminate both explicit and implicit entrepreneurial and managerial barriers	Deleted
		Leaders obtain supportive resources for business transformation and innovation both within and outside the company	Deleted
		Leaders often provide employees with support and help to reduce barriers in work	
		Leaders actively establish an atmosphere of innovation	
		1. Leaders strive for employee appreciation of business innovation and transformation.	
		Leaders often encourage employees to realize individual values via work	
		Leaders actively structure work teams to facilitate employee cooperation	
		Leaders can inspire employees to accomplish the business goals	
		Leaders have a clear understanding of the business scope, what to do and what not to do	Deleted
		Leaders clearly define the limits of company ability and avoid unnecessary resource consumption	Deleted
		Leaders are good at integrating human and material resources to carry out the work within the company capacity	
		Leaders have strong confidence in employees accomplishing fixed tasks	
		Leaders often encourage employees to innovate	
		Leaders make quick and effective operational decisions according to company capacity and resources	
Turnover intention	Liang (1999)	I often want to leave this company	
		I’m highly likely to find a new job next year	
		I often want to change my job recently	
Affective commitment	Yao et al. (2008)	I’m glad to work in this company	
		I am a part of this company	
		I feel a sense of belonging in this company	
		I have a great affection for this company	
Person-job fit	Weng and McElory (2010)	I think this job fits me very well	
		I’m equipped with necessary experience, skills and knowledge for this job	
		Current work condition fits my demands	
		My personality fits this job well	
